# Pteropods counter mechanical damage and dissolution through extensive shell repair

**DOI:** 10.1038/s41467-017-02692-w

**Published:** 2018-01-17

**Authors:** Victoria L. Peck, Rosie L. Oakes, Elizabeth M. Harper, Clara Manno, Geraint A. Tarling

**Affiliations:** 1 British Antarctic Survey, Natural Environment Research Council, High Cross, Madingley Road, Cambridge, CB3 0ET UK; 2 Department of Geosciences, The Pennsylvania State University, University Park, PA 16802 USA; 3 Academy of Natural Sciences of Drexel University, 1900 Benjamin Franklin Parkway, Philadelphia, PA 19103 USA; 4 Department of Earth Sciences, University of Cambridge, Downing Street, Cambridge, CB2 3EQ UK

## Abstract

The dissolution of the delicate shells of sea butterflies, or pteropods, has epitomised discussions regarding ecosystem vulnerability to ocean acidification over the last decade. However, a recent demonstration that the organic coating of the shell, the periostracum, is effective in inhibiting dissolution suggests that pteropod shells may not be as susceptible to ocean acidification as previously thought. Here we use micro-CT technology to show how, despite losing the entire thickness of the original shell in localised areas, specimens of polar species *Limacina helicina* maintain shell integrity by thickening the inner shell wall. One specimen collected within Fram Strait with a history of mechanical and dissolution damage generated four times the thickness of the original shell in repair material. The ability of pteropods to repair and maintain their shells, despite progressive loss, demonstrates a further resilience of these organisms to ocean acidification but at a likely metabolic cost.

## Abstract (web summary)

Sea butterflies, or pteropods, are often presented as being at threat from ocean acidification on account of their fragile shells being susceptible to dissolution. Here the authors show that pteropods are able to perform extensive repair to damaged shells, suggesting they may not be as vulnerable as previously thought.

## Introduction

The current rate of anthropogenic carbon release is unprecedented during the past 66 million years^1^, leading to acidification of the ocean through heightened uptake of CO_2_ into surface waters. In addition to decreasing pH values, ocean acidification diminishes the availability of carbonate ions, the building blocks of calcium carbonate, making calcification of shells and skeletons in marine organisms more energetically demanding and unprotected shells susceptible to dissolution^2^. Polar oceans are predicted to be among the first to become undersaturated with respect to carbonate^3, 4^ and calcifying organisms living in the polar regions, especially those which make their shell from aragonite, the more soluble form of calcium carbonate, have been the focus of studies investigating the likely impact of ocean acidification. While polar benthic molluscs exhibit resilience to ocean acidification^5^, microscopic pelagic molluscs, pteropods, have been shown to decrease their rate of calcification^6–8^ and exhibit shell dissolution within waters undersaturated with respect to aragonite, Ω_Ar_ ≤ 1^2, 9, 10^. In a similar way to paint on a car protecting the metal body from exposure to the corrosive effects of the atmosphere, shelled molluscs, including pteropods, have an organic coating known as the periostracum protecting the shell from dissolution upon exposure to undersaturated waters^11, 12^. Observations of rapid pteropod shell dissolution have been accounted for by the thin periostracum covering pteropod shells allowing dissolution to occur beneath it^13^. However, a recent study finding that pteropods exposed to seasonally undersaturated waters were only vulnerable to shell dissolution where the periostracum had been damaged^14^ opened debate on the effectiveness of the pteropod periostracum^15, 16^. Accepting the notion that intact pteropod periostracum is effective in impeding dissolution, once damaged, for example, scratched by a predator or weakened by microbes, the underlying shell structure is no longer protected and exposure of aragonite to waters of Ω_Ar_ ≤ 1 can lead to progressive shell dissolution^14^. If dissolution breaches the shell wall, the animal’s ability to regulate buoyancy and internal chemistry is compromised and the soft body becomes vulnerable to infection and predation^17^. Internal repair of failed predation, endolith boring and other physical and chemical damage to the shell is common in molluscs through the secretion of carbonate onto the inner wall of the shell^18–22^. While previous studies have indicated that internal thickening of the shell is likely to be common to pteropods^8, 14^, the extent to which this process can protect these animals from the deleterious effects of exposure to undersaturated waters in the natural environment has not been observed.

In this study, we use micro-computed tomography (micro-CT) (cf. ref. ^23^) to analyse damaged shells of polar species *Limacina helicina* collected within the Fram Strait. Shell thickness measurements across both pristine and damaged areas of shell reveal that specimens are able to maintain shell integrity by extensive thickening of the inner shell wall. The ability of *L. helicina* to repair shell damage in naturally undersaturated conditions indicates that this species has more potential to counteract the deleterious effects ocean acidification may have on their shells than previously considered.

## Results

### Damaged shells within Fram Strait sea ice

**Fig. 1:**
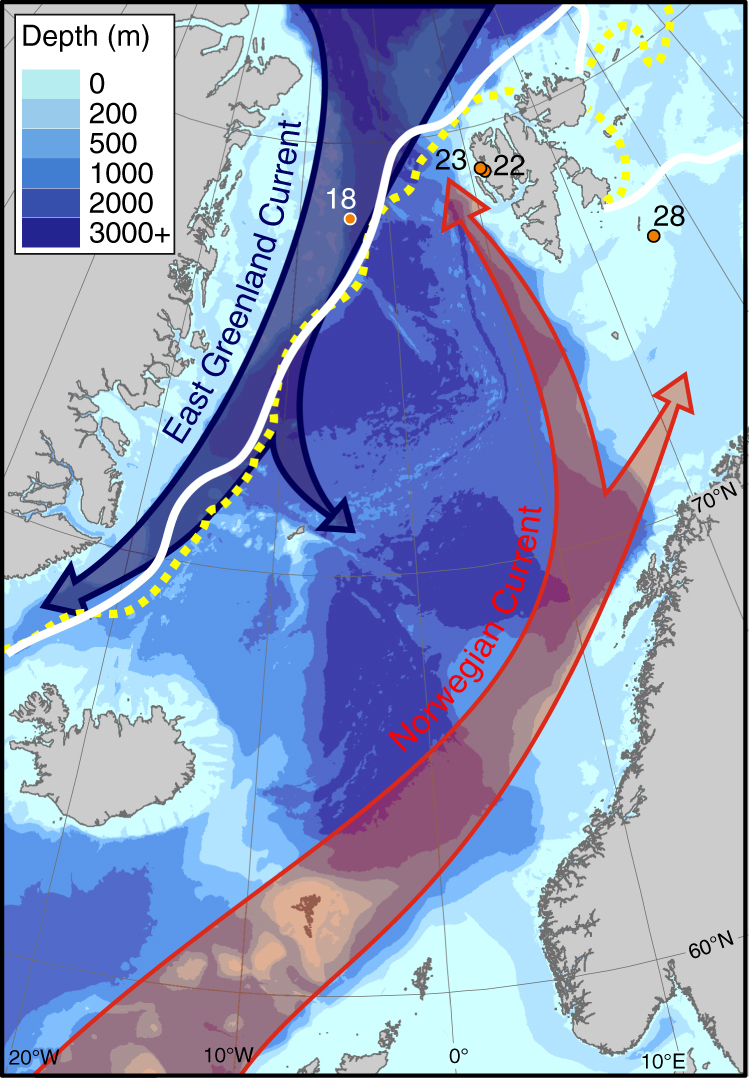
Location of sites where *L. helicina* were collected in June 2012. Solid white line indicates the maximum sea ice extent in the preceding winter (March 2012 average). Dashed yellow line indicates the average position of the sea ice edge during June 2012 when the samples were collected. The East Greenland Current, flowing south out of the Arctic Ocean, is represented by a blue arrow. Atlantic waters, carried by the Norwegian Current, are represented by the red arrow. Bathymetry used is The GEBCO_2014 Grid, version 20150318, www.gebco.net. Sea ice extent data from the National Snow and Ice Data Centre^39^

We analyse specimens of polar species *L. helicina* recovered during research expedition JR271 during June 2012^14^. Of the 28 stations where the British Antarctic Survey’s motion-compensated bongo net was deployed, *L. helicina* were recovered at four localities, associated with some of the coldest waters encountered during the cruise (Supplementary Table 1; Supplementary Figs. 1, 2). At one of these stations (Station 18; Fig. 1), within sea ice in Fram Strait, a third (13) of the recovered pteropods (*n* = 36) exhibited notable shell damage not observed at the other sites (Supplementary Fig. 3). Three of the specimens exhibiting shell damage presented a history of trauma to the shell surface that caused deep damage in the inner whorls and exposure of the shells’ internal microstructure.

### Multi-image analysis determines the stages of damage and repair

**Fig. 2:**
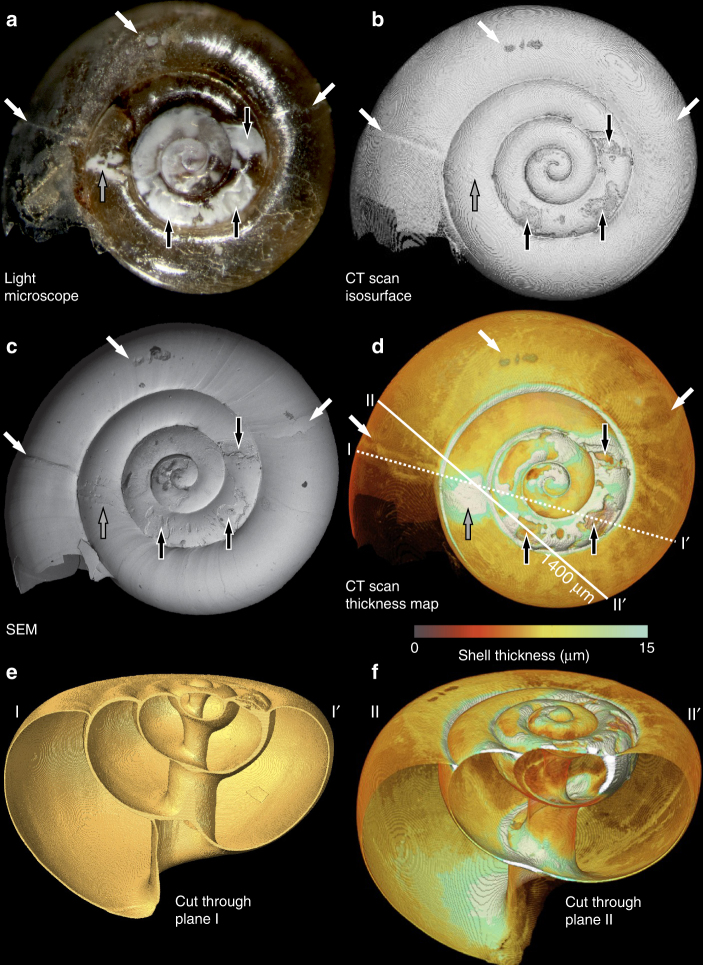
Comparison of imaging methods of *L. helicina* specimen to demonstrate shell thickening in response to surface damage and dissolution. **a** Light microscopic image; **b** isosurface rendering of the surface of the shell; **c** SEM; **d** CT-scan thickness map; **e**, **f** cut-through images along planes I–I’ and II–II’ indicated in **d**, respectively. The shell thickness colour scale used in **d** and **f** is indicated within the range 0–15 μm. Shell exceeding a thickness of 15 μm appears white. Black arrows indicate areas of deep surface damage in the inner whorls with evidence of dissolution and associated shell thickening observed in SEM and CT images, respectively. Grey arrow indicates shell fractures in the penultimate whorl with no evidence of dissolution but associated shell thickening observed. White arrows indicate fractures and mechanical damage in the final whorl with no evidence of dissolution and no associated shell thickening observed

**Fig. 3:**
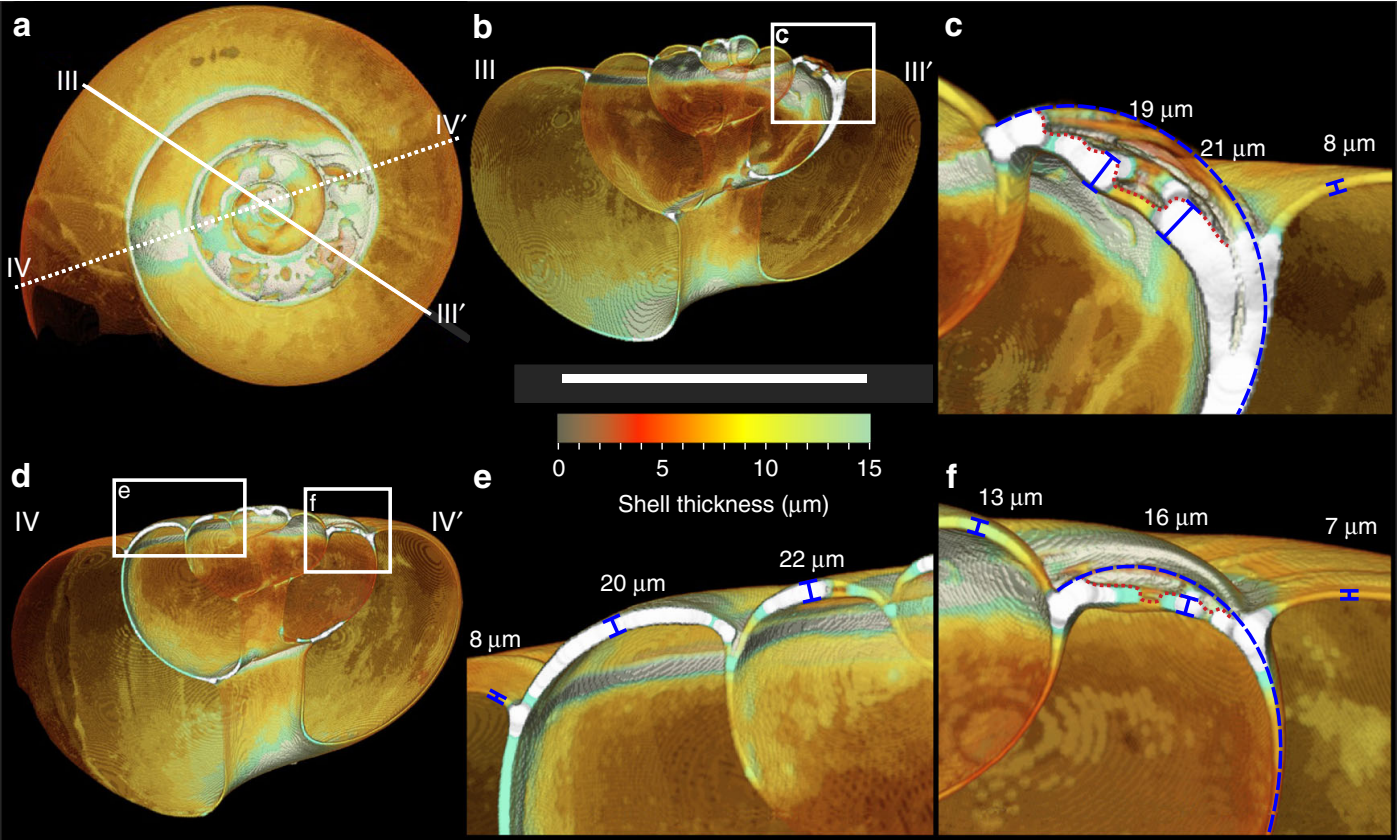
Analysis of cross-sections to determine the extent of surface damage/dissolution and repair calcification. **a** CT-scan thickness map; **b** cross-section along plane III-III’ shown in **a**; **c** zoom-in on area indicated in **b**; **d** cross-section along plane IV–IV’ shown in **a**;** d** and **e** zoom-ins on areas indicated in **d**. White scale bar represents 1 mm. The shell thickness colour scale is indicated within the range 0–15 μm. Shell exceeding a thickness of 15 μm appears white. Point thickness measurements are indicated by blue barred lines (perpendicular to the outer surface). In **c** and **f**, the blue dashed line indicates the position that the original shell surface would have occupied prior to damage, based on the profile of the undamaged shell of the same whorl in the background. The red dotted line indicates the current outer surface of the shell along the plane of the cross-section

**Fig. 4:**
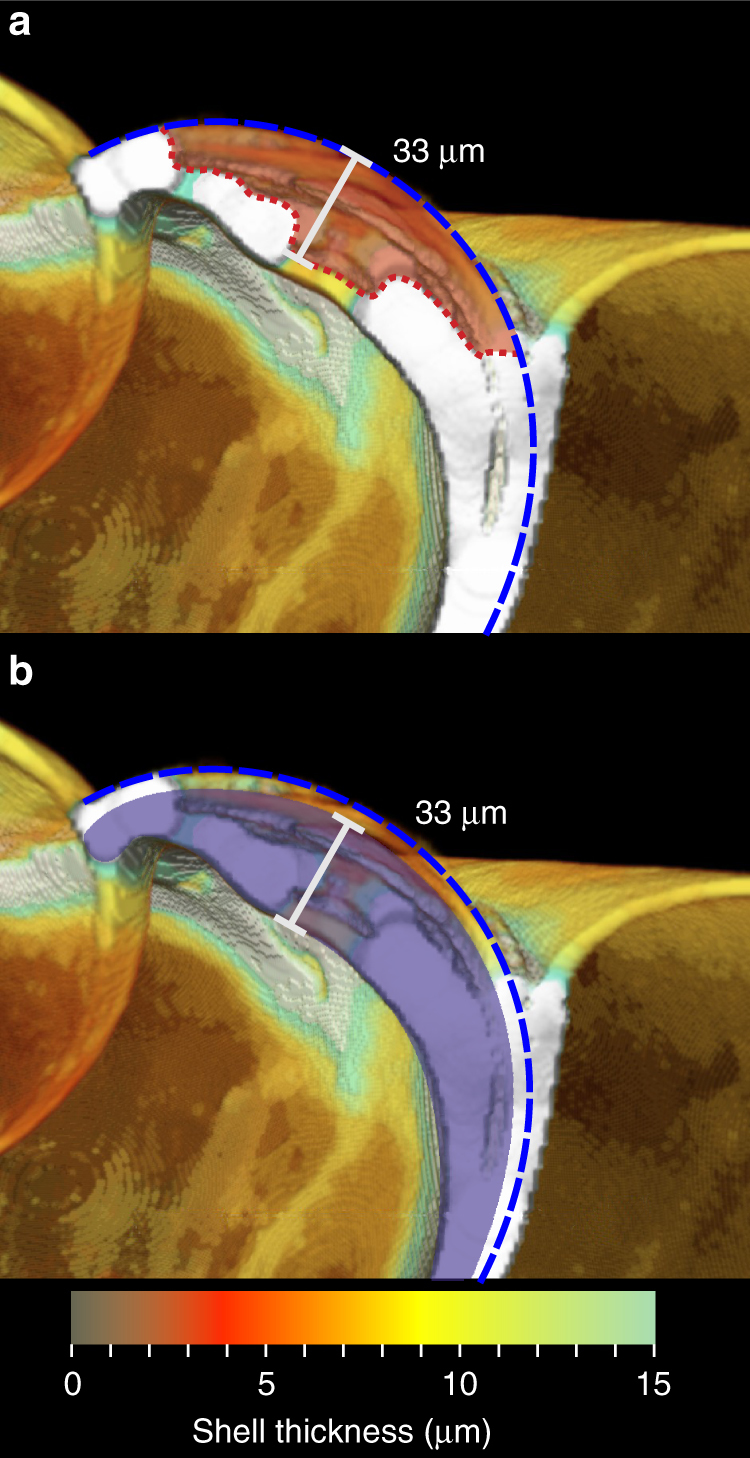
Assessment of shell loss and thickening. Cross-section as Fig. 3c with blue dashed line indicating the original shell surface prior to damage/dissolution. **a** The red shaded area indicates shell thickness lost between the original shell surface and the current shell surface (red dotted line). **b** The blue shaded area indicates shell thickening. Assuming an original shell thickness of 8 μm, this specimen has thickened the shell to a maximum of 33 μm at this location

Comparison of light microscopic, scanning electron microscopic (SEM) and CT scans (Fig. 2; but also see Supplementary Figs. 5 and 6) reveal a range of shell damage with varying stages of repair. Pristine shell appears translucent under light microscope (Fig. 2a), with smooth-surfaced subtle growth bands evident in SEM (Fig. 2c). There is little variability in the thickness of pristine shell, typically measuring 7–8 μm (Fig. 2d; also ref. ^24^). Anomalies within otherwise pristine shell include areas of deep outer surface damage (examples highlighted with black arrows in Fig. 2) within the inner whorls. The isosurface image (Fig. 2b) clearly highlights areas where the outer surface of the shell has been lost such that the current shell surface is recessed from the pristine shell surface. Cross-sections reveal that the depth of the deepest areas of damage exceeds the thickness of pristine shell (Fig. 3c, f) confirming that the original shell has been completely lost^14^. The absence of a perforation in the shell, exposing the animal within, indicates that additional shell material has been secreted to 'patch up' the damaged area from the inside. This modification to the inner shell wall is readily evident, with areas adjacent to deep surface damage frequently over two times thicker than pristine shell (Fig. 3c, f). The site of deepest damage in the illustrated specimen is 33 μm below the original shell surface (Fig. 4a). Assuming an original shell thickness of 8 μm, this specimen generated over 4 times this thickness in repair material at this site of damage (Fig. 4b).

A further area of notable shell thickening is highlighted with a grey arrow in the penultimate whorl, corresponding to opacity under light microscope (Fig. 2). Despite this opacity, the SEM image reveals that there is no notable dissolution of the shell’s outer surface (no aragonite rods exposed), but there are two relatively 'clean' historic fractures, possibly the result of more recent failed predation. It appears that the animal has built up shell material beneath these fractures effectively to weld the shell back together. The lack of surface dissolution but opacity in light microscope coincident with the area of shell thickening could indicate that areas of shell repair can also appear opaque in light microscope, perhaps on account of a different microstructure.

Within the final whorl, there is no evidence of surface dissolution or shell thickening; however, mechanical fractures and surface damage are evident, suggesting that dissolution and the repair response have not yet been initiated (Fig. 2; white arrows).

## Discussion

In many cases, initial damage to the shell is likely to have been mechanical in nature, on account of scratch-like features possibly indicative of failed predation attempts (Supplementary Fig. 4). The exposure of individual aragonite rods evident under SEM, however, is consistent with ‘etching’ observed following deliberate experimental exposure of pteropods to undersaturated waters^2, 9, 10^. We therefore propose that aragonite exposed at sites of damage had been susceptible to dissolution by undersaturated waters secondarily, exacerbating the mechanical shell damage further. Continued exposure to undersaturated waters is the most plausible explanation for the extensive loss of repair material from the outer surface, necessitating the persistent thickening of the inner shell, at sites of deep damage (Fig. 4). The progressive loss of shell in these areas resulted from the inability of the animal to replace the periostracum (ref. ^11^ and references therein), meaning that repair material remained unprotected and was subject to progressive dissolution on the outer surface. Despite this continued shell loss, the animal appears to have invested energy into maintaining a minimum shell thickness comparable with that of pristine shell. While the sub-sea-ice waters from which these specimens were collected had the lowest Ω_Ar_ values encountered during the cruise, they were not undersaturated^25^ (Supplementary Fig. 2). We are not aware of any physical property or carbonate chemistry measurements beneath the sea ice in Fram Strait in the winter months that can validate our hypothesis that these specimens were exposed to undersaturated waters, but we do note that waters beneath sea ice in comparable, seasonal sea ice regions within the Arctic do become undersaturated during the winter months. The most comparable study area we found was in the Amundsen Gulf, where Fransson et al.^26^ measured under-ice-water conditions through the formation and subsequent melt of seasonal (first year) sea ice over winter 2007–2008. Ω_Ar_ was observed to decrease beneath the sea ice with minimum values (0.6–1.4) recorded in March/April 2008. Sea ice melt began in May and enhanced sea–air exchange and photosynthesis, drawing down pCO_2_, caused Ω_Ar_ to increase to values of 1.4–2. These Ω_Ar_ values recorded in the Amundsen Gulf in May are similar to those measured in waters beneath melting ice within the Fram Strait in June (Supplementary Table 1, Supplementary Figure 2). If we assume a similar seasonal range of Ω_Ar_ beneath sea ice east of Greenland and the Amundsen Gulf, we can anticipate that waters at and around Station 18 were likely to have been close to saturation (Ω_Ar_ ~ 1) or undersaturated (Ω_Ar_ < 1) over the winter of 2011–2012. Alternatively, these specimens may have been advected by the East Greenland Current from undersaturated waters in the Arctic Ocean. Given the evidence of shell dissolution and the likelihood of these specimens having been exposed to undersaturated waters within their life history, we consider shell damage to result from a combination of mechanical damage and subsequent dissolution of exposed aragonite.

To understand the time lines involved in shell damage, dissolution and the internal repair/thickening response, we consider the life cycle of *L. helicina* within Fram Strait. In this example, the pteropod would have been recruited during the spring/summer of 2011 and overwintered beneath the sea ice before collection in the summer of 2012^14^. We propose that the specimen survived at least one predation attempt, causing mechanical damage to the surface of the shell, before or during the winter months. We anticipate that Ω_Ar_ decreased beneath the sea ice^25^ during the winter period accounting for the extensive, localised dissolution in areas of shell where aragonite had been exposed by mechanical damage. *L. helicina* are observed to suppress linear shell growth when food is scarce during the winter months^10, 27^. Consistent with Lischka & Riebesell^27^, we propose that overwinter calcification efforts were focussed on repair and maintenance of the shell from the inside. In spring time, we anticipate that photosynthesis and enhanced air–sea exchange (following sea ice melt) would have increased Ω_Ar_^26^, reducing the progressive dissolution of exposed aragonite, allowing the animal to relax energy expended on shell maintenance. At the same time, enhanced food availability would have fuelled a surge in linear shell growth^10, 27^, most likely represented by the near-pristine final whorl in our case study example (Fig. 2). The areas of fracture and mechanical damage on the last whorl would have only been vulnerable to dissolution during the following sea ice season when waters again became undersaturated, with extensive dissolution triggering the repair response.

Unlike specimens recovered from Fram Strait, *L. helicina* recovered from the outer region of Kongsfjord and the Barents Sea (Stations 22, 23 and 28) did not exhibit deep shell damage. However, shells collected at these stations do exhibit mechanical damage^14^, which is consistent with our assumption that all populations would be exposed to comparable predatory pressures. The absence of shell opacity or ‘etching’ associated with historic fractures (Supplementary Fig. 3), however, indicates that, dissimilar to the Fram Strait specimens, dissolution at sites of mechanical damage did not occur to specimens from outer-Kongsfjord and the Barents Sea, suggesting that these specimens had not been exposed to undersaturated water. At the time of collection, Ω_Ar_ exceeded 2 at Stations 22, 23 and 28. While waters at Station 18 (Fram Strait) were also oversaturated with respect to aragonite at the time of collection, we speculate that exposure to undersaturated waters likely occurred beneath sea ice during the preceding winter. We are confident that the specimens collected within Fram Strait overwintered on account of their shell diameter averaging 1255 ± 146 μm. The shells of winged specimens (non-veligers) collected from outer-Kongsfjord and the Barents Sea had an average diameter of 753 ± 145 μm and 600 ± 139 μm, respectively. The shell diameters of specimens from Stations 22, 23 and 28 are smaller than the >900 μm shell diameter typical of overwintering specimens studied within at NyAlesund^10, 27^; however, we propose that it is more plausible that the outer-Kongsfjord and Barents Sea specimens were spawned in 2011 rather than just a few weeks prior to collection coincident with the spring bloom^28^. Despite the likelihood that specimens recovered from Stations 22, 23 and 28 had overwintered, we find little evidence to support them having been exposed to undersaturated waters. Although Ω_Ar_ < 1 was observed at 200 m in the mid-fjord during February 2010^10^, waters in outer-Kongsfjord^29^ and the Barents Sea^30^ remain supersaturated with respect to aragonite during the winter months, consistent with the absence of dissolution damage observed in shells collected from these locations. The contrast in saturation state between Station 18 (Fram Strait) and the outer-Kongsfjord and Barents Sea stations can largely be attributed to regional oceanography and sea ice conditions (Fig. 1). The warm Atlantic waters transported within the Norwegian Current have a high total alkalinity^31^, which Comeau et al.^32^ considered to explain the relatively low sensitivity of western Svalbard waters to ocean acidification. The near absence of sea ice during the winter months at Stations 22, 23 and 28 further accounts for persistently supersaturated waters throughout the year. In contrast, the East Greenland Current, exporting Arctic waters, is cold with a low saturation state^33^. In addition, the presence of sea ice at Station 18 further modifies carbonate chemistry in sub-sea ice waters, decreasing Ω_Ar_ over the winter months^26,^ meaning Station 18 was likely close to saturation or undersaturated with respect to aragonite during the winter of 2011/2012. We conclude that the populations of *L. helicina* most susceptible to dissolution in areas of shell damage within the Greenland-Norwegian Arctic region are those in true polar waters, with sea ice playing a key role in decreasing the saturation state.

Sea ice seasons are doubly troublesome for these polar pteropods since exposed aragonite is progressively dissolved at the same time that food is scarce. In order to maintain the integrity of the shell during winter exposure to undersaturated waters, the rate of internal repair calcification would need to, at least, keep pace with the rate of dissolution (Fig. 4). Metabolic upregulation of overwintering *L. helicina* suggests that repair calcification is energetically demanding^27^, and we recognise the possibility that there may be a diminishing limit to the amount of damage that an animal is able to repair under further undersaturated conditions. While the extent of undersaturation, duration of exposure and incidence of periostracal damage are variables that may test the tolerance of *L. helicina* in this environment, the availability of food remains a key factor in driving the metabolic effort^34^. Considering that food is scarce over the winter months, we assume that stored energy reserves are used to fuel overwinter shell repair^27, 35^. Continuing reduction in sea ice cover will impact the quantity and timing of food availability in this region^36^, which may benefit the ability of this species to balance their energy budgets^35^ over a shorter period of food scarcity. However, any benefit that a reduced sea ice season may offer will ultimately become redundant when light intensity becomes the limiting factor on controlling the onset of spring blooms, meaning *L. helicina* living in this environment will always endure periods of extreme food shortage as undersaturation becomes more pervasive^4^.

We note that it is unfortunate that the number of individuals we recovered exhibiting extensive damage and repair is so few. The restricted sample size is due to both the limited occurrence of pteropod populations with a history of likely exposure to undersaturated waters encountered along the cruise track and restricting time dedicated to zooplankton sampling when pteropods were present. However, the restricted sample size should not detract from the clear demonstration that *L. helicina* in the natural environment is able to thicken the shell wall and maintain shell integrity when the shell is damaged. *L. helicina* populations in environments where the incidence of shell damage is high and waters are, at least seasonally, undersaturated with respect to aragonite warrant further investigation and monitoring to better assess the impact and tolerances of *L. helicina* to undersaturated conditions, particularly during the winter months.

Our findings add to growing evidence that many polar calcifiers, exposed to undersaturated waters, can withstand and repair damage to their shells^37^, perhaps on account of natural exposure to heightened physical and chemical variability, which have resulted in organisms developing or exhibiting an inherent resilience strategy (cf. ref. ^38^). We do not claim that *L. helicina* will be immune to ocean acidification on account of their ability to maintain their shells, but propose efforts should shift to assessing the metabolic cost of repair calcification when predicting the tolerance of this species to future environmental conditions.

## Methods

### Specimen collection and storage

Cruise JR271 consisted of 28 science days dedicated to obtaining a quantitative understanding of the impact of ocean acidification on the surface ocean biology and ecosystem and on the role of the surface ocean within the Arctic (https://www.bodc.ac.uk/resources/inventories/cruise_inventory/report/11432/).

A comprehensive suite of routine sampling and measurements performed at stations each day prescribed a strict schedule for instrument deployment. Zooplankton collection on research expedition JR271 was primarily based on routine deployment of a motion-compensated Bongo net at 28 morning stations. The net (100 and 200 μm mesh) was deployed between 0 and 200 m depth (water depth permitting) three times in succession (with the exception of sites where *L. helicina* was found where additional deployments were made where possible; see Supplementary Table 1), with one set of samples being immediately preserved (100 μm net sample preserved within ethanol and 200 μm net in formalin for other analyses) and the two (or more) sets of samples used for hand picking copepods, pteropods and foraminifera for incubation or analysis. Once picked from the sample, specimens were rinsed in buffered milli-Q water on collection and air dried in individual wells within specimen slides. Dried specimens were imaged under light microscope to be catalogued and provide a means of assessing any post-collection damage to the shells. Shipboard images of *L. helicina* collected during JR271 are available (Supplementary Fig. 3).

### Light microscope and SEM imaging

No additional preparation of the specimens was performed prior to light microscope or low-vacuum SEM imaging (Supplementary Fig. 4). For high-vacuum SEM imaging (Fig. 2, Supplementary Fig. 3) and CT scanning, specimens were sputter coated with ~10 nm of gold-palladium.

### CT scanning

Two specimens were scanned on a phoenix v|tome|x m at 65 kV and 230 µA for 52 min at GE in Lewistown, PA. The resolution of this scan was 1 µm. One specimen was scanned on a FEI Quanta 650 SEM equipped with a Gatan XμM module at 22 kV, spot 6, camera exposure 200μs for one frame at the Natural History Museum, London. The resolution of this scan was 0.6 µm. Data were processed and visualised using *VG Studio Max 2.2, Avizo 9.1*, and *ImageJ*. Shell thickness was analysed using the *BoneJ* plug in for *ImageJ*. The resultant data were converted into greyscale (*Greys* lookup table) and exported as a .tiff stack. Shell thickness maps were created from this data in *Avizo 9.1* using the VolRen thickness colour scheme set to a range of 0–0.015 mm.

### Data availability

All data are available from the authors upon request.

## Electronic supplementary material

Supplementary Information
