# Supplementary material for: Pteropods counter mechanical damage and dissolution through extensive shell repair
Source: Nat Commun. 2018 Jan 17;9:264. doi: 10.1038/s41467-017-02692-w (PMC5772362; doi:10.1038/s41467-017-02692-w)
Supplement: Supplementary file 1 — Supplementary Information [file 41467_2017_2692_MOESM1_ESM.pdf]

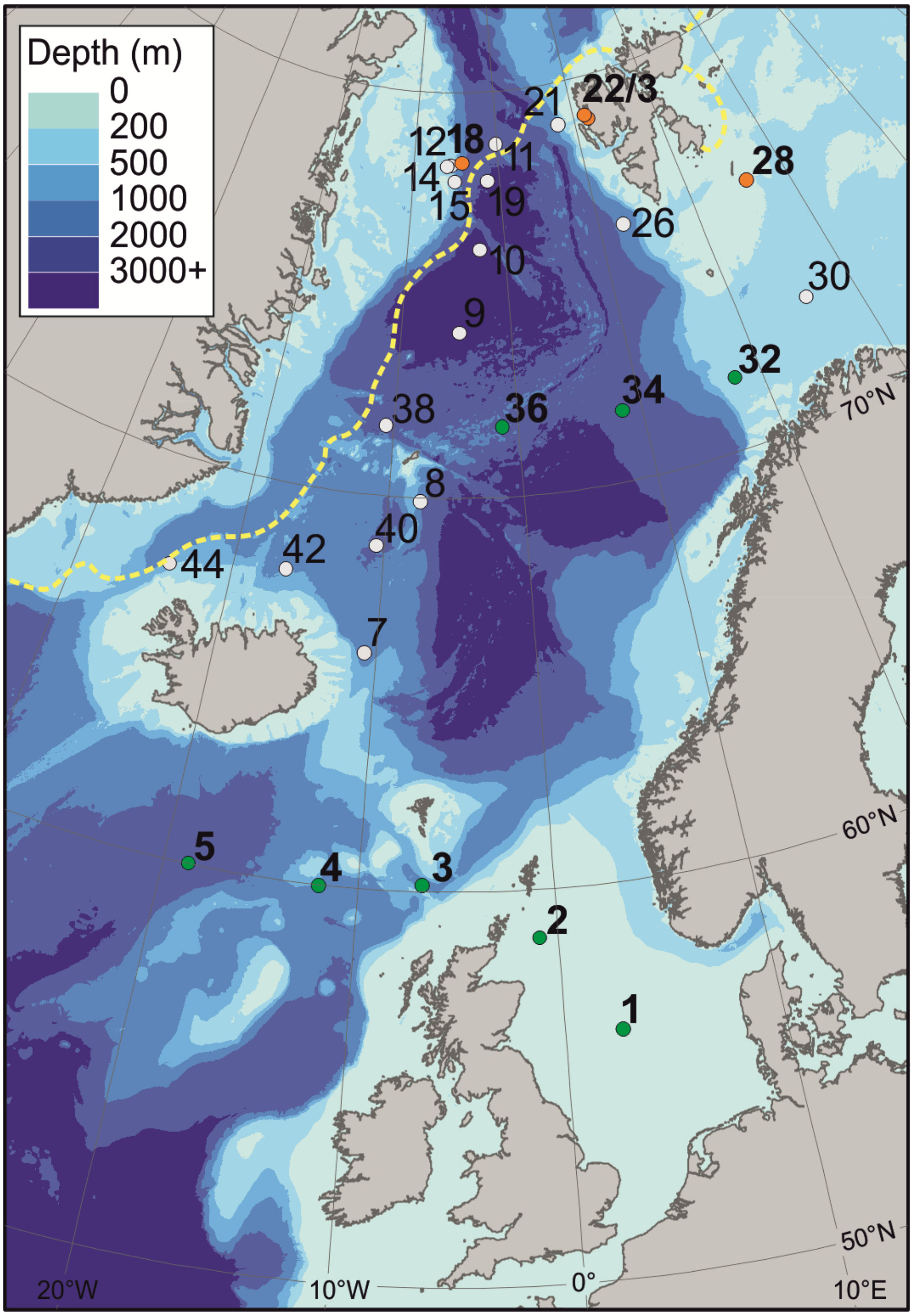

Supplementary Figure 1. **Stations where bongo nets were deployed to recover pteropods during JR271.** White symbols indicate stations where no pteropods were recovered, orange symbols indicate recovery of *L. helicina* and green symbols indicate recovery of *L. retroversa*. Stations numbered as cruise report https://www.bodc.ac.uk/resources/inventories/cruise_inventory/report/11432/ and Supplementary Table 1. Yellow dashed line indicates average sea ice extent during July 2012 (https://nsidc.org/data/seaice_index/archives.html)[1]. Station symbol colours consistent with coloured bars in Supplementary Figure 2. Bathymetry used is The GEBCO_2014 Grid, version 20150318, www.gebco.net.

Date	Station	Latitude	Longitude	Pteropods recovered			Number of net deployments at station	Water properties at 6 m water depth[1]		
				None	*L. retroversa*	*L. helicina*		SST (°C)	Salinity	ΩAr
03/06/2012	1	56.2666	2.6332		✓		3	11.0	35.1	2.5
04/06/2012	2	58.7398	-0.8615		✓		3	10.3	35.3	2.8
05/06/2012	3	60.1340	-6.7042		✓		3	10.6	35.4	2.4
06/06/2012	4	59.9713	-11.9781		✓		3	10.6	35.3	2.8
07/06/2012	5	60.0014	-18.6703		✓		3	10.2	35.2	2.4
10/06/2012	7	65.9794	-10.7183	✓			3	4.6	34.8	2.8
11/06/2012	8	69.8957	-7.5771	✓			3	4.1	35.1	2.0
12/06/2012	9	74.1165	-4.6930	✓			3	1.1	34.8	1.7
13/06/2012	10	76.1753	-2.5495	✓			3	1.8	34.9	1.8
14/06/2012	11	78.7181	0.0041	✓			3	1.8	34.6	2.4
**15/06/2012**	**12**	**78.2394**	**-5.5575**	✓			**3**	**-1.6**	**32.1**	**1.4**
**16/06/2012**	**14**	**78.2157**	**-6.0063**	✓			**3**	**-1.6**	**32.3**	**1.2**
17/06/2012	15	77.8309	-5.0311	✓			3	1.7	34.4	2.4
**18/06/2012**	**18**	**78.2886**	**-4.2655**			✓	**4**	**-1.6**	**32.5**	**1.4**
19/06/2012	19	77.8425	-1.3159	✓			3	2.7	34.3	2.8
20/06/2012	21	78.9826	7.9800	✓			3	6.1	35.0	3.0
20/06/2012	22	78.9557	11.9248			✓	4	5.1	34.2	2.4
21/06/2012	23	79.0582	11.4384			✓	5	3.7	34.2	2.5
22/06/2012	26	76.2620	12.5418	✓			3	5.8	35.0	2.1
23/06/2012	28	76.1595	26.0616			✓	3	1.0	34.5	2.1
24/06/2012	30	72.8916	26.0017	✓			3	6.3	35.1	2.1
25/06/2012	32	71.7520	17.9008		✓		3	7.8	35.0	2.4
26/06/2012	34	71.7475	8.4428		✓		3	6.9	35.1	2.2
27/06/2012	36	71.7453	-1.2673		✓		3	6.0	35.1	2.2
28/06/2012	38	71.7484	-10.5971	✓			3	3.4	34.1	1.9
29/06/2012	40	68.6951	-10.5760	✓			3	4.9	34.9	2.0
30/06/2012	42	67.8304	-16.4218	✓			3	7.0	34.9	2.6
01/07/2012	44	67.2623	-24.0362	✓			3	3.6	33.1	2.3

Supplementary Table 1. Stations during cruise JR271 where bongo nets were deployed to recover pteropods. Stations in bold are stations within sea ice and the highlighted station is the station (within sea ice) where *L. helicina* were recovered exhibiting deep damage to their shells. Note that additional net deployments were permitted at Stations 18, 22 and 23 to optimise recovery of specimens of *L. helicina*.

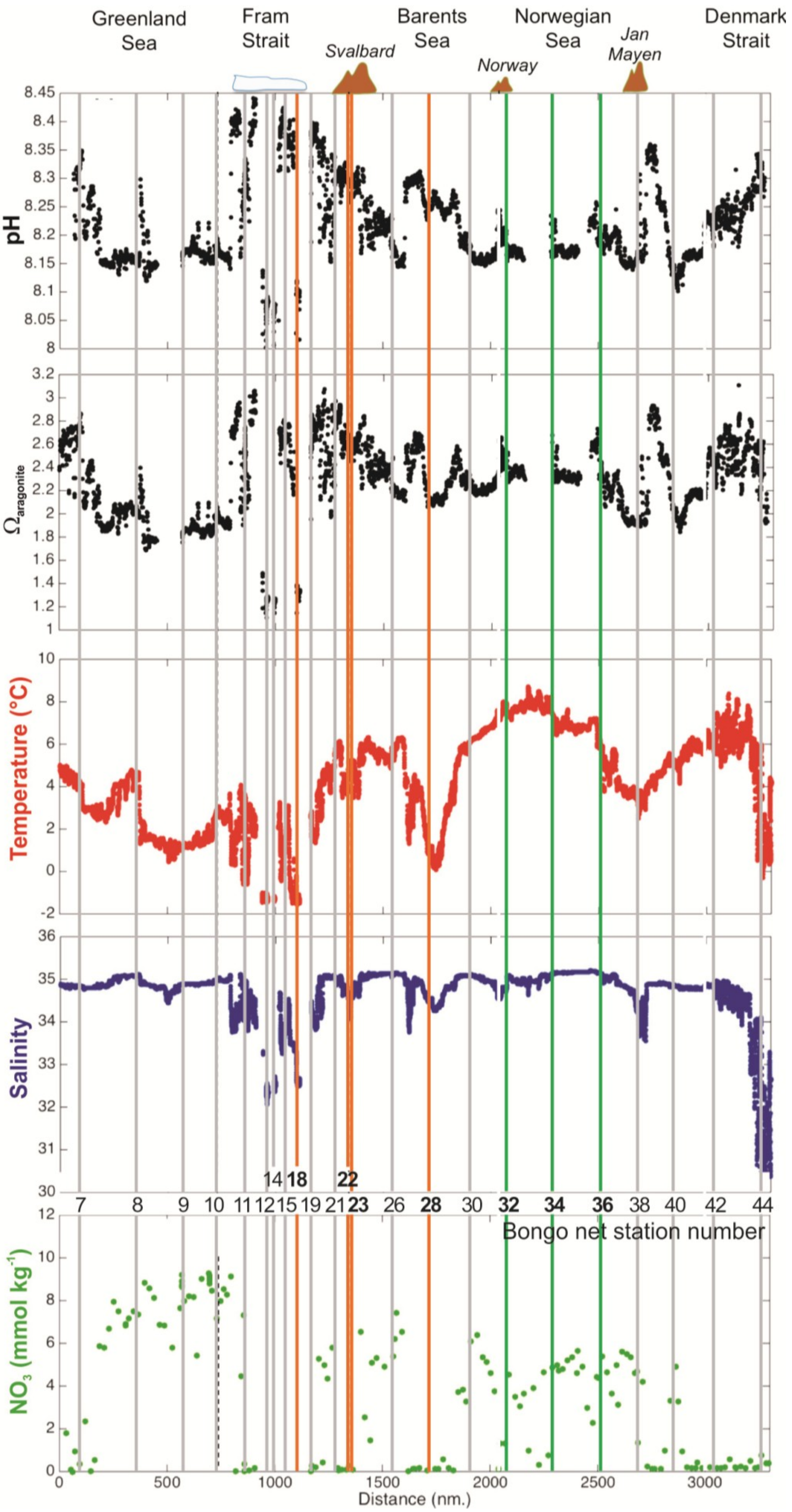

Greenland Sea
Fram Strait
Svalbard
Barents Sea
Norway
Norwegian Sea
Jan Mayen
Denmark Strait
pH
$\Omega_{aragonite}$
Temperature (°C)
Salinity
Bongo net station number
7 8 9 10 11 12 14 15 18 19 21 22 23 26 28 30 32 34 36 38 40 42 44
NO_3 (mmol kg^{-1})
Distance (nm.)

Supplementary Figure 2. **Variations in pH, temperature, salinity and nitrate concentrations in surface waters (6 m) along the cruise track of JR271 and incidence of bongo nets deployed to recover pteropods**. Figure adapted from Tynan *et al.*[2]. Coloured vertical bars indicate Bongo net deployments to recover pteropods within the Greenland, Barents and Norwegian Seas. Station numbers detailed at top of NO_3 plot. Grey bars indicate stations where bongo nets were deployed but no pteropods were recovered, orange bars (with bold number) indicate recovery of *L. helicina* and green bars (with bold number) indicate recovery of *L. retroversa*. Note lowest Ω_{Ar} values are recorded beneath sea ice in Fram Strait coincident with Stations 12, 14 and 18.

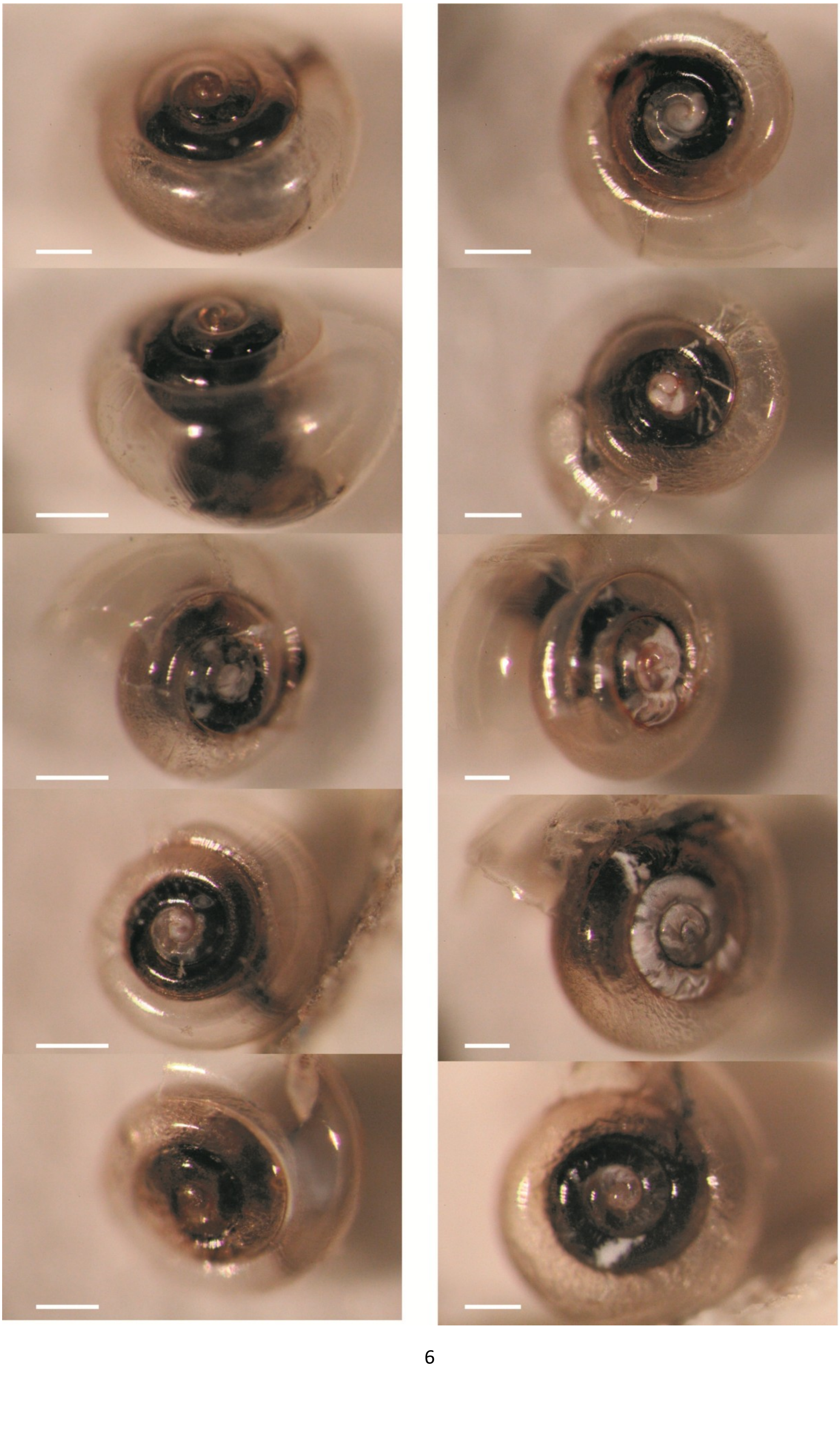

Station 18

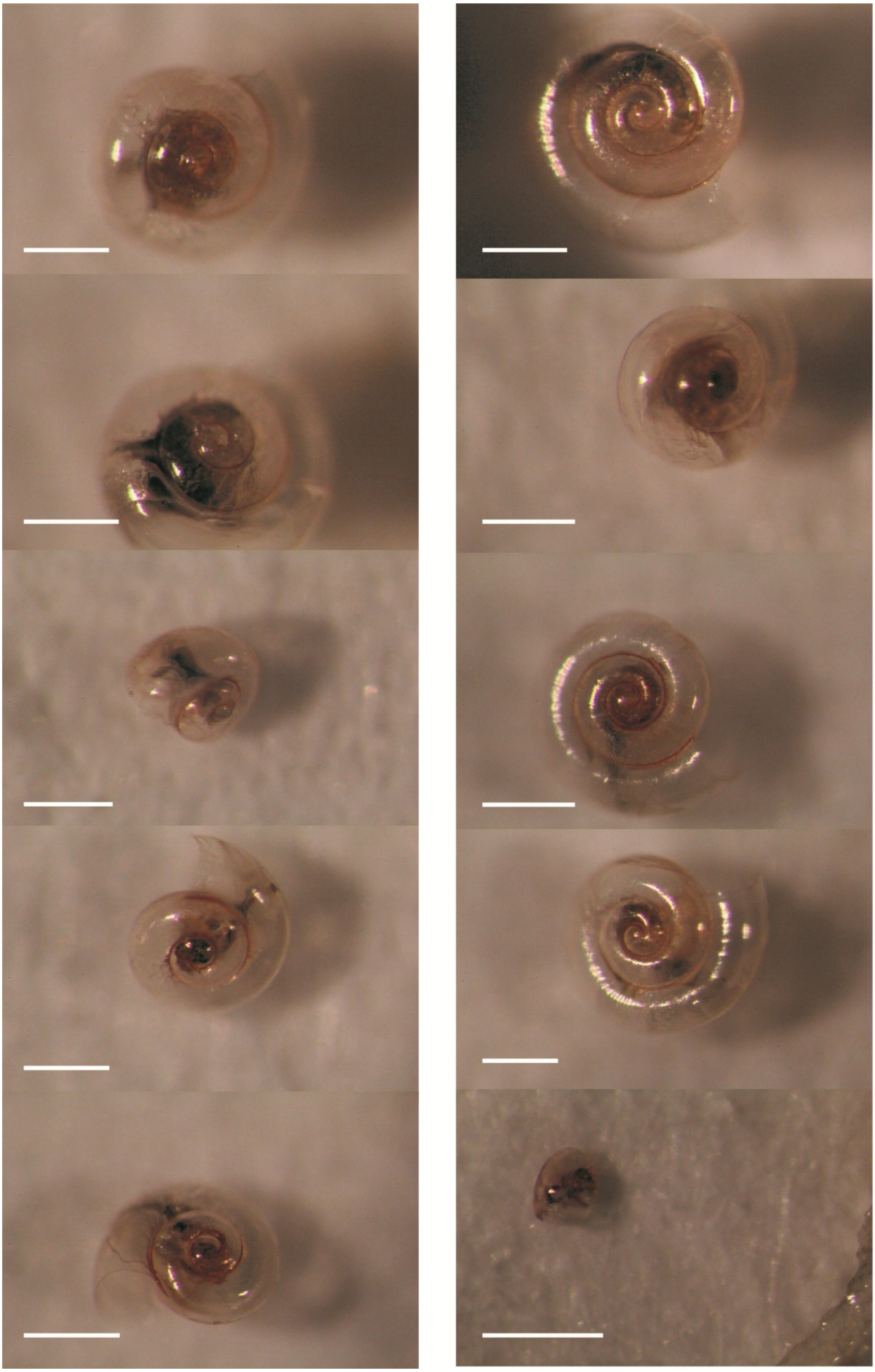

Station 23

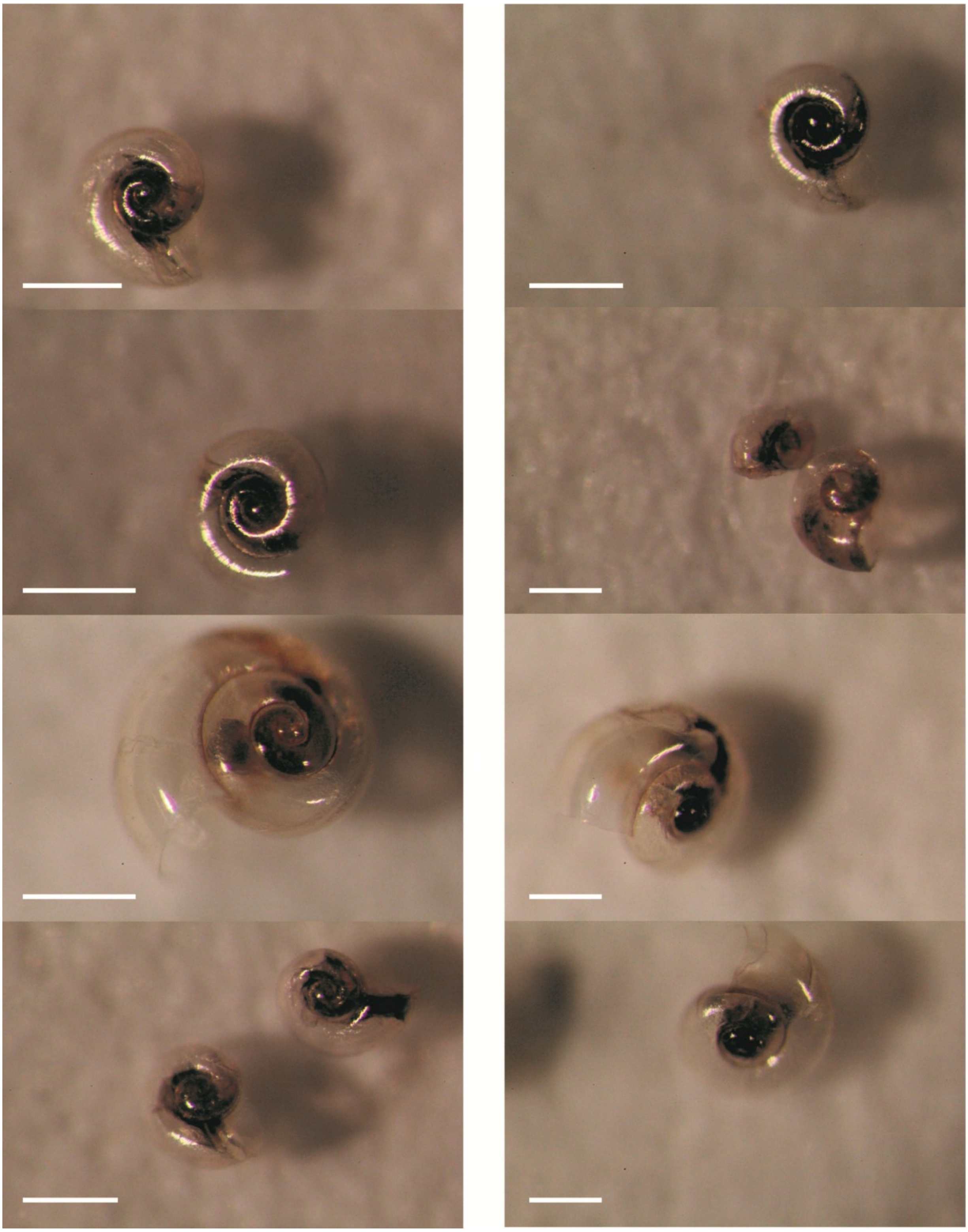

Station 28

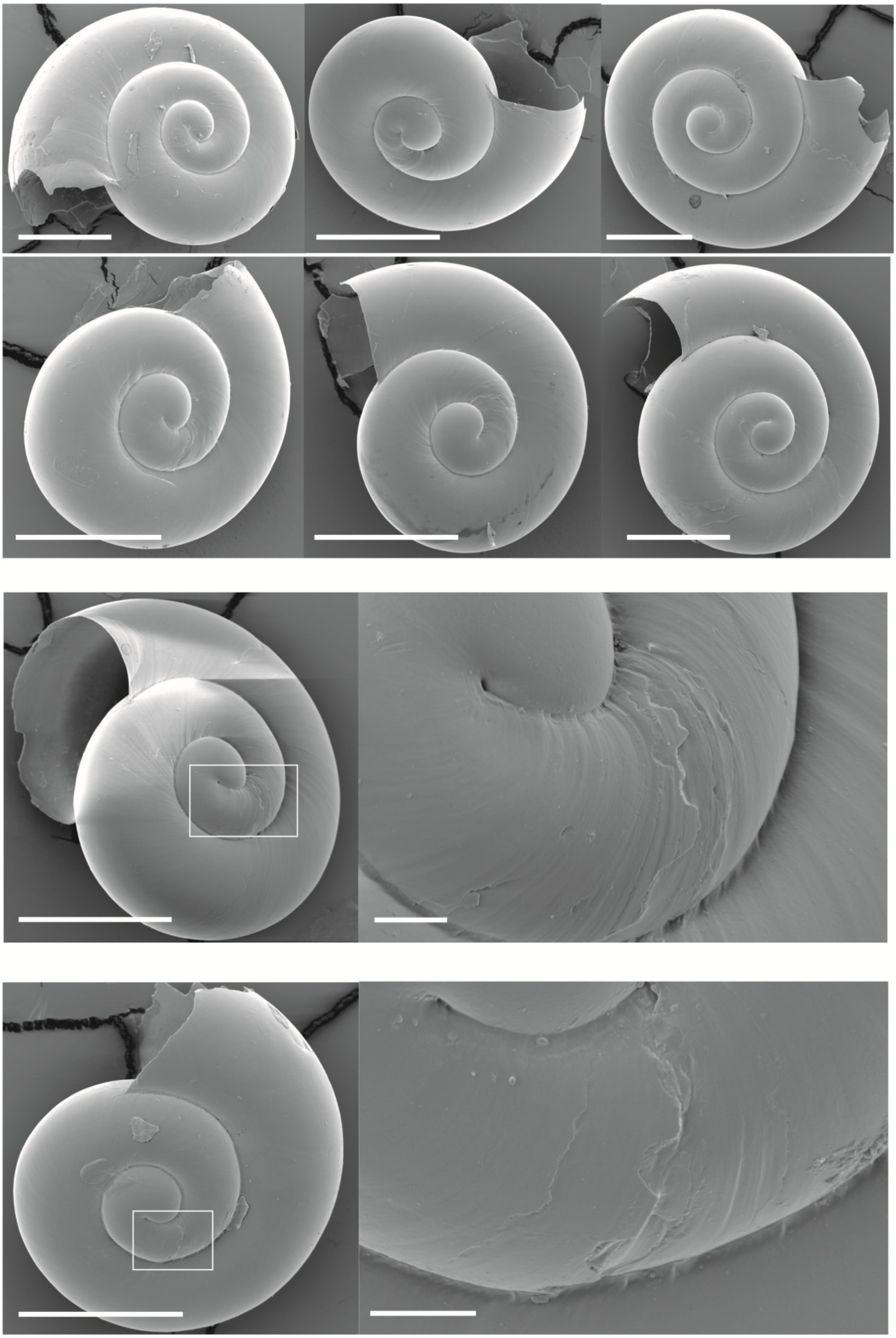

Supplementary Figure 3. Photographs of a sub-set of specimens of *L. helicina* collected at Stations 18, 23 and 28 and SEM images of specimens from Stations 23 and 28. Light microscope photographs were taken on-board the ship as a means of cataloguing each sample and documenting any change in the condition of the shells during transport and storage. Images were taken after the specimens has been rinsed in buffered milli-Q water and dried in an oven (35-40 °C) for 12 hours. Scale bar 300 μm in each light microscope image. Note that it is only specimens collected at Station 18 that exhibit localised opacity within their shells, with deep damage evident in two specimens shown here.

SEM images of specimens from Stations 23 and 28 confirm that despite a comparable incidence of mechanical damage (fractures) to the shells as observed at Station 18, the absence of "etching" indicates that areas of mechanical damage were not susceptible to subsequent dissolution. Scale bars in SEM images 200 μm and 20 μm in high-magnification inserts.

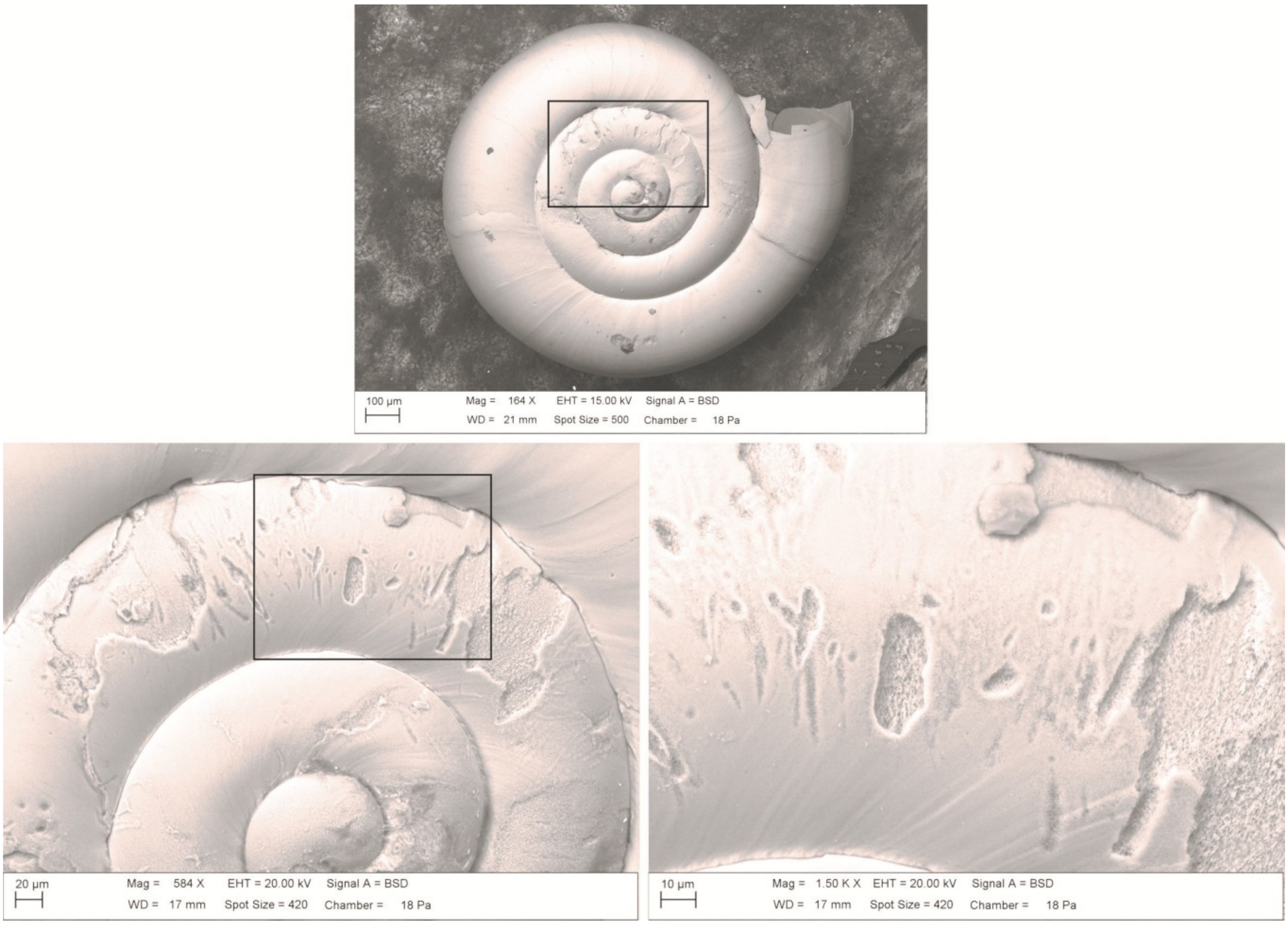

Supplementary Figure 4. Demonstration that shell damage discussed here is historic and not an artefact of collection. While mechanical damage to these delicate shells is to a routine occurrence during collection, these low vacuum (un-coated) SEM images demonstrate that the deep damage to the inner whorls occurred within the lifetime of the animal and not as an artefact of collection. Note that the subsequent whorl to that which was heavily damaged forms around the areas of deep damage indicating that damage to the inner whorl preceded growth of the outer whorl. Damage to the aperture occurred post-collection during specimen handling.

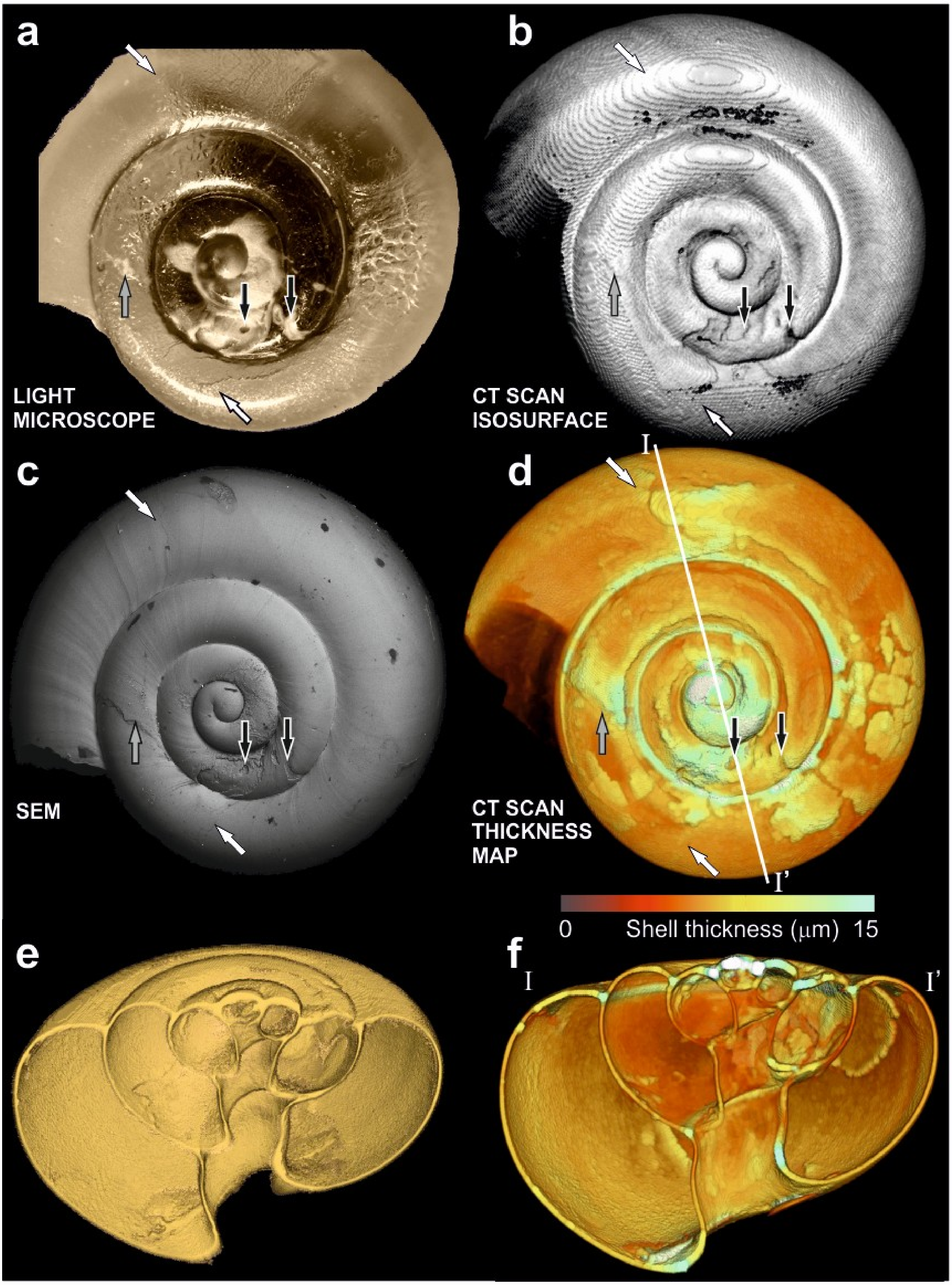
a
LIGHT MICROSCOPE
b
CT SCAN ISOSURFACE
c
SEM
d
CT SCAN THICKNESS MAP
I
I'
0 Shell thickness (μm) 15
e
f
I
I'

Supplementary Figure 5. Comparison of imaging methods of a further L. helicina specimen exhibiting extensive shell thickening/repair in response to deep surface damage/dissolution within inner whorls. a. light microscope image; b. isosurface rendering of the surface of the shell; c. SEM; d. CT-scan thickness map; e and f show cut-through images along plane I-I' indicated in d. The thickness colour scale used in d and f is indicated within the range 0-15 μm. Shell exceeding a thickness of 15 μm appears white.

Black arrows indicate areas of deep surface damage in the inner whorls with evidence of secondary dissolution and associated shell thickening observed in SEM and CT-images respectively. Grey arrow indicates shell fractures in the penultimate whorl with no evidence of dissolution but associated shell thickening observed. White arrows indicate fractures and mechanical damage in the final whorl with no evidence of dissolution and no associated shell thickening observed.

Residual body matter adhered to the inner wall of the final whorl is evident in d and f and should not be confused with thickening of the shell wall.

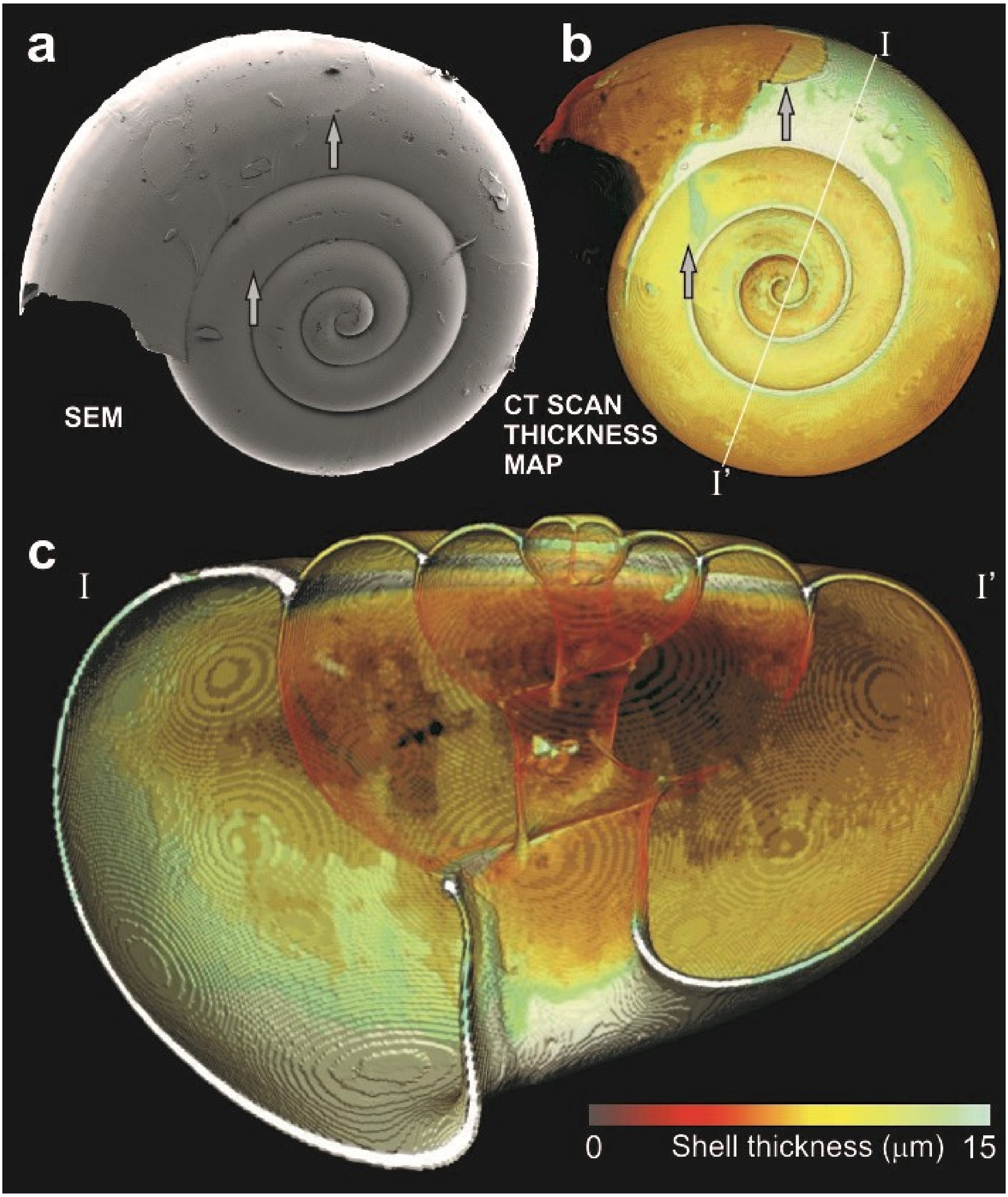

Supplementary Figure 6. SEM and CT-scans of specimen exhibiting a no deep surface damage within the inner whorls. Note the consistent thickness of the shell wall within the inner whorls where the SEM indicates no deep surface damage or fracture. Grey arrows indicate shell fractures evident within the penultimate and final whorl which have not been subject to dissolution on the outer surface yet thickening/repair of these relatively recent areas of damage is evident in the CT-scan thickness map and cross section.

Supplementary References

1. Fetterer, F., Knowles, K., Meier, W., Savoie, M. & Windnagel, A. K. updated daily. *Sea Ice Index, Version 3*. [Indicate subset used]. Boulder, Colorado USA. NSIDC: National Snow and Ice Data Center. doi: http://dx.doi.org/10.7265/N5K072F8. (2017) [Accessed 01 June 2017].

2. Tynan, E *et al*. Physical and biogeochemical controls on the variability in surface pH and calcium carbonate saturation states in the Atlantic sectors of the Arctic and Southern Oceans. Deep Sea Res. **127**, 7-27 (2016) https://doi.org/10.1016/j.dsr2.2016.01.001
